# Phylogeny and Taxonomical Investigation of *Trichoderma* spp. from Indian Region of Indo-Burma Biodiversity Hot Spot Region with Special Reference to Manipur

**DOI:** 10.1155/2015/285261

**Published:** 2015-01-28

**Authors:** Th. Kamala, S. Indira Devi, K. Chandradev Sharma, K. Kennedy

**Affiliations:** Institute of Bioresources and Sustainable Development, Ministry of Science & Technology, Government of India, Takyelpat Institutional Area, Imphal, Manipur 795001, India

## Abstract

Towards assessing the genetic diversity and occurrence of *Trichoderma* species from the Indian region of Indo-Burma Biodiversity hotspot, a total of 193 *Trichoderma* strains were isolated from cultivated soils of nine different districts of Manipur comprising 4 different agroclimatic zones. The isolates were grouped based on the morphological characteristics. ITS-RFLP of the rDNA region using three restriction digestion enzymes: Mob1, Taq1, and Hinf1, showed interspecific variations among 65 isolates of *Trichoderma*. Based on ITS sequence data, a total of 22 different types of representative *Trichoderma* species were reported and phylogenetic analysis showed 4 well-separated main clades in which *T. harzianum* was found to be the most prevalent spp. among all the *Trichoderma* spp. Combined molecular and phenotypic data leads to the development of a taxonomy of all the 22 different *Trichoderma* spp., which was reported for the first time from this unique region. All these species were found to produce different extrolites and enzymes responsible for the biocontrol activities against the harmful fungal phytopathogens that hamper in food production. This potential indigenous *Trichoderma* spp. can be targeted for the development of suitable bioformulation against soil and seedborne pathogens in sustainable agricultural practice.

## 1. Introduction

The genus* Trichoderma *was widely studied due to its rapid growth, capability of utilizing diverse substrates, and resistance to noxious chemicals [1].* Trichoderma* are often the predominant components of the mycoflora in soils of various ecosystems, such as agricultural fields, prairie, forest, salt marshes, and desert [2]. Several* Trichoderma* species are significant biocontrol agents against fungal phytopathogens and act as stimulators for plant health [3, 4].* Trichoderma* species produce diverse metabolites, most notably commercially important cellulose, hemicellulases, antibiotics, peptaibiotics, and the toxins (such as Trichodermamides) and Trichothecenes that display* in vitro* cytotoxicity [5–8].

Due to the ecological importance of* Trichoderma* spp. and its application as a biocontrol agent in the field, it is important to understand its biodiversity and biogeography. However, accurate species identification based on morphology is difficult because of the paucity and similarity of morphological characters [9, 10] and increasing numbers of morphologically cryptic species [11, 12]. This has already resulted in incorrect identification [13]. Therefore, with the advent of molecular methods and identification tools based on sequence analysis of multiple genes, it is now possible to identify every* Trichoderma* isolate and recognize it as a putative new species [9, 14, 15]. The current diversity of the holomorphic genus* Hypocrea*/*Trichoderma* is reflected in approximately 160 species, the majority of which have been recognized by molecular phylogeny of pure cultures and herbaria specimens [15, 16].

The natural mechanisms promoting high fungal diversity have remained unclear, but it seems likely that differential preference for soil and climatic conditions and host plants play the key role [17]. A series survey of* Trichoderma* spp. was conducted in different regions such as China by Zhang et al., 2005 [18], Siberia and Himalayas by Kullnig et al., 2000 [19], Egypt by Gherbawy et al., 2004 [20], and Central and South American region by Druzhinina et al., press. Their studies led to the identification of several new species [21, 22] and furthermore revealed a unique species in all these regions. This could be the result of geographic/climatic bias of some species. In this study we intended to determine the occurrence and species diversity of* Trichoderma* collected from unique biodiversity hotspot region of NE India.

## 2. Materials and Methods

### 2.1. Geography of Sampling Sites

**Figure 1:**
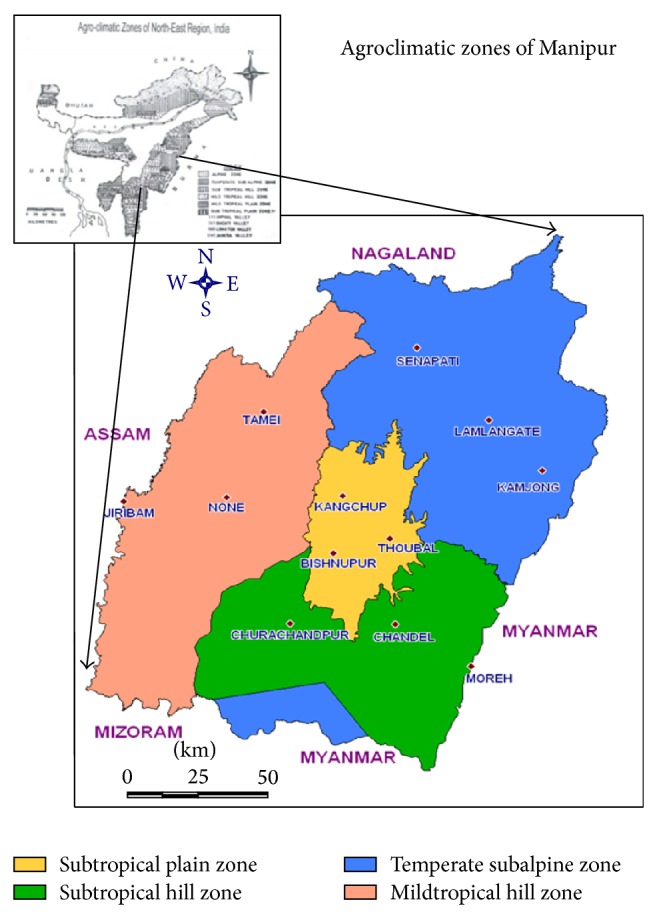
Map of Manipur showing different agroclimatic conditions.

Sampling was done from nine different districts of Manipur comprising four distinct agroclimatic zones, namely, (i) subtropical plain zone, (ii) subtropical hill zone, (iii) temperate sub-Alpine zone, and (iv) midtropical hill zone, which differ in their geographic location, altitude, and climate (Figure 1). Subtropical plain zone comprises Imphal West (711 m above sea level; average rainfall, 1259.5 mm), Imphal East (790; 1413.0 mm), Thoubal (790 m; 1318.39 mm); Bishnupur (828 m; 1204.2 mm), and some portion of Senapati district (2500 m; 671–1454 mm). Subtropical hill zone comprises Churachandpur (1764 m; 3080 mm) and Chandel district (787 m; 1650.00–3430.85 mm). Temperate sub-Alpine zone comprises Senapati and Ukhrul district (1338 m; 1763.7 mm). While midtropical hill zone comprises Ukhrul and some portion of Imphal and Tamenglong district (1260 m; 3135 mm).* Trichoderma* isolates investigated in this study were isolated from the total 90 soil samples collected from nine different districts of Manipur (10 samples from each district pooled from 5 spots).

### 2.2. Isolation and Storage of Pure Cultures

Rose Bengal agar [23] was used as a selective medium for the isolation of* Trichoderma* species, using soil dilution plating method. 1lt. of* Trichoderma* selective medium comprises MgSO_4_7H_2_O (0.2 g), K_2_HPO_4_ (0.9 g), KCl (015 g), NH_4_NO_3_ (1.0 g), Glucose (3.0 g), Rose Bengal (0.15 g), and agar (20 g). Putative* Trichoderma* colonies were purified by two rounds of subculturing on potato dextrose agar (PDA). Pure cultures were maintained in mineral oil at 4°C and also at −20°C by suspending the fungal spores in 10% (w/v) skim milk incorporated with silica powder. At the same time the cultures were lyophilized and stored in ampules.

### 2.3. Morphological Analysis

For morphological analysis, strains were grown on PDA at 27°C for 7-8 days. Growth rates were determined at 20, 25, 30, 35, and 40°C for 72 h on PDA [24]. Microscopic observations were done using trinocular microscope (Carl Zais Axio ImageM2, Germany). Conidiophore structures and morphology were examined on macronematous conidiogenous pustules or from fascicles when conidia were matured. Conidial morphology and size were recorded after 14 days of incubation.* Trichoderma* species were identified according to Gams and Bissett [25] and Samuels et al. [26, 27].

### 2.4. DNA Extraction and Amplification

The extraction of genomic DNA was performed with minor modification as described by Hermosa et al. [28]. The ITS region of the nuclear small-subunit rRNA gene was amplified in an automated thermocycler (Bio-rad-C1000 Thermal Cycler) using the primers ITS1 (TCCGTAGGTGAACCTGCGG) and ITS4 (TCCTCCGCTTATTGATATGC) as described by White et al., 1990 [29]. The PCR reactions were performed in a total volume of 50 *μ*L, containing 1x standard PCR incubation buffer, 0.5 *μ*M of each primer, 200 *μ*M of each of the four deoxyribonucleotide triphosphates, 1.25 U Taq polymerase and 20 ng genomic DNA with the PCR condition of 94°C for 1 min, annealing at 52°C for 60 sec and 90 sec elongation at 74°C and final extension of 7 min at 74°C in 30 cycles. A negative control with all the reaction mixtures except the DNA template was included with each set of the PCR amplification reactions. Prior to digestion, a 10 *μ*L aliquot of each PCR product, together with the 100 bp ladder (Biogene) which acts as a reference marker, was resolved by gel electrophoresis on 1.5% resolution agarose gel for 40 min at 70 V. Finally, the PCR products were visualized under UV light using Gel imaging system (BioRad, Chemi Doc, MP).

### 2.5. Restriction Digestion

For restriction fragment length polymorphism (RFLP) analysis, PCR products of 65* Trichoderma* isolates were digested with three restriction enzymes, namely, Taq1, Hinf1, and Mbo1 in 20 *μ*L reaction mixtures consisting of 10x buffer (2.0 *μ*L), enzyme (0.4 *μ*L), PCR product (6.0 *μ*L), and MQ water (11.6 *μ*L). Reaction mixtures of Mbo1 enzyme were incubated for 1 hr at 37°C, Taq1 for 1 hr at 65°C, and Hinf1 for 7 hrs at 37°C. The RFLP bands were separated by 2.5% (w/v) agarose gel electrophoresis stained with ethidium bromide. ITS-RFLP data were recorded by scoring all DNA bands and compiled in a binary matrix.

### 2.6. Sequence Assembly and Alignment

Amplification products obtained from PCR reactions with unlabeled ITS primers (ITS1 and ITS4) were used for sequencing and DNA sequences obtained from each forward (ITS) and reverse (ITS4) primer were inspected individually for quality. The consensus sequences of each strain were obtained using Gene Runner software Version 3.05 (Hastings software Inc. Hasting, NY, USA; http://www.generunner.net/). All sequences were aligned using Clustal W with default settings [30].

### 2.7. Phylogenetic Analysis

Sequence alignment was conducted with the CLUSTAL W program [31]. All characters were equally weighted and alignment gaps were treated as missing data. Relative support for specific clades represented in the tree was estimated by bootstrap analysis of 1000 replicates [32]. Nucleotide divergences were estimated using Kimura's two-parameter method. Sequence data analysis was carried out by a stepwise approach.

### 2.8. Extrolites

Culture extracts were made from the potato dextrose broth medium. The extracts were analysed by HPLC and GC-MS data. Authentic analytical standards were employed for retention time and retention index comparison with the extrolites detected.

### 2.9. Enzyme Production

Screening of chitinase activity was performed using chitin detection medium according to the method given by Agarwal and Kotasthane [33]. Chitin detection medium comprises (all amounts are per litre) 4.5 g of colloidal chitin, 0.3 g of MgSO_4_7H_2_O, 3.0 g of (NH_4_)SO_4_, 2.0 g of KH_2_PO_4_, 1.0 g of citric acid monohydrate, 15 g of agar, 0.15 g of bromocresol purple, and 200 mL of Tween-80; pH adjusted to 4.7. Protease activity of* Trichoderma* isolates was determined using skim milk agar medium [34]. For screening of *β*-1,3-glucanases activity, medium amended with laminarin was used according to the modified method given by El-Katatny et al., 2001 [35].

## 3. Results

A total of 193* Trichoderma* spp. were isolated from the cultivated soil of Manipur using Rose Bengal agar medium. This region consists of 4 different agroclimatic conditions with varied soil types and topographical identity (Table 1 and Figure 1). Out of the total collection, 65* Trichoderma* isolates were selected based on their morphological identification and their genomic DNA was amplified using the ITS 1 and 4 primers of the rDNA region.

### 3.1. ITS-RFLP

**Figure 2:**
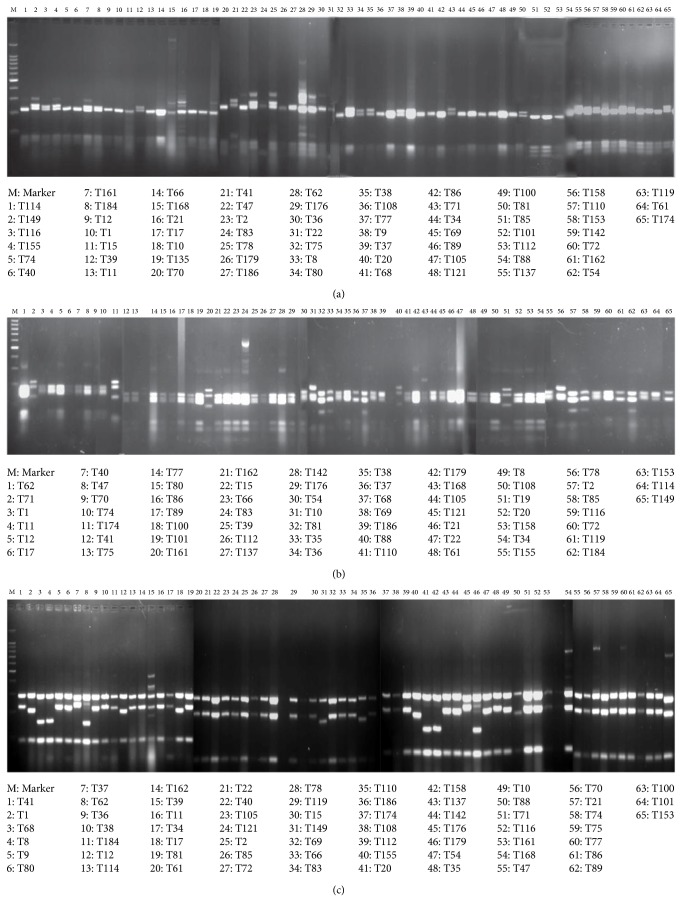
Restriction digestion of 65* Trichoderma* isolates: (a) Taq1, (b) Mbo1, and (c) Hinf1.

The phylogenetic diversity of 65* Trichoderma* strains was analyzed using ITS restriction fragment length polymorphism (ITS-RFLP) of the ribosomal spacer (rDNA) region. The amplified rDNA fragment length ranged from 548 to 607 bp. The ITS region provided greater resolution for distinguishing isolates of* Trichoderma* spp. ITS-RFLP carried out by using MboI and Hindf1 illustrated distinct bands to differentiate among the groups as compared to the samples treated with TaqI (Figure 2).* Trichoderma harzianum* and* Trichoderma aureoviride* exhibited a high level of intraspecific polymorphism.

### 3.2. Phylogenetic Inference

**Figure 3:**
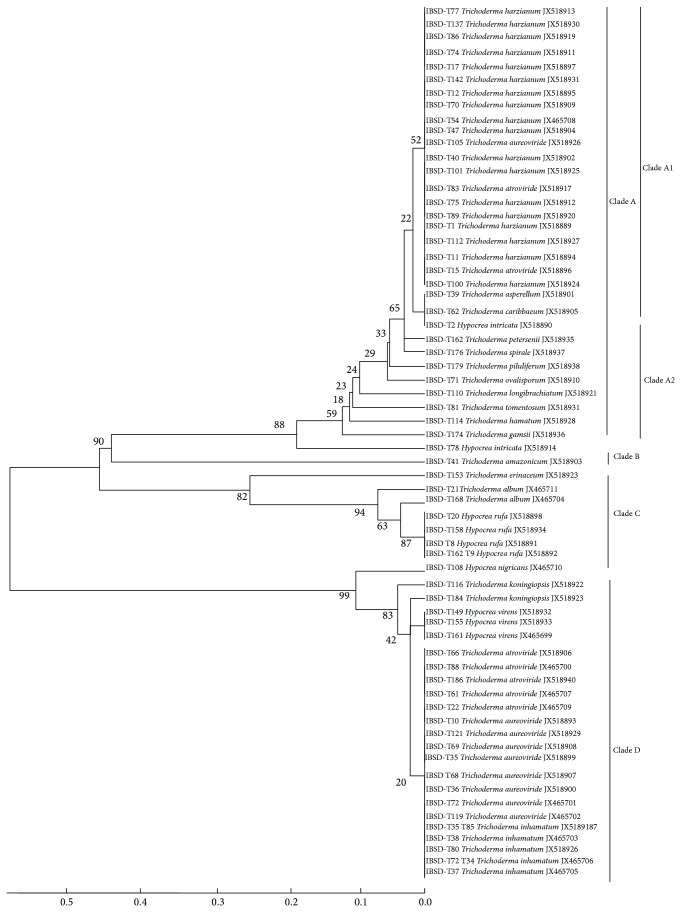
Phylogenetic tree of the 65* Trichoderma* isolates inferred by maximum parsimony analysis of ITS1 and ITS 4 sequences. The numbers given over branches indicate bootstrap coefficient.

The phylogenetic tree obtained by sequence analysis of ITS region of 65* Trichoderma* strains is represented in Figure 3. The ITS sequence was chosen for this analysis because it has been shown to be more informative with various sections of the genus* Trichoderma* [36, 37]. A maximum parsimony analysis of the alienable ITS-sequences of the 65* Trichoderma* strains demonstrated a total of 4 distinct clades and all the clades were phylogenetically distinct from each other. Clade A comprises mainly* T. harzianum* representing the occurrence of biggest group of* Trichoderma* spp. which was supported by a bootstrap value of 88%. Further clade A is divided into 2 subclades A1 and A2. A1 comprises* T. harzianum*,* T. aureoviride*,* T. asperellum*,* T. caribbaeum,* and* H. intricata*. A2 comprises* T. petersenii*,* T. spirale*,* T. piluliferum*,* T. ovalisporum*,* longibrachiatum*,* T. tomentosum*,* T. hamatum*,* T. gamsii,* and* H. intricata,* whereas clade B had close match with* T. amazonicum* species supported by a bootstrap value of 90%.* T. amazonicum* was unique as it formed a separate branch basal. Clade C represents four strains of* H. rufa* along with* T. erinaceum*,* T. album,* and* H. nigricans* supported by a bootstrap value of 82%, whereas clade D comprises mainly of* T. aureoviride*,* T. atroviride*,* T. koningiopsis*,* H. virens,* and* T. inhamatum* with bootstrap value of 99%.

### 3.3. Species Identification

**Figure 4:**
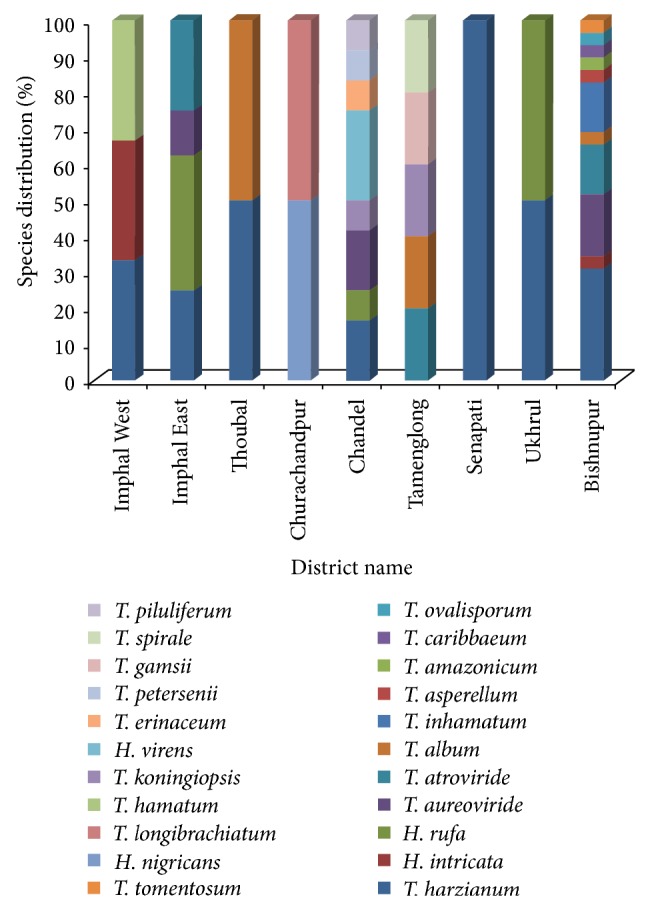
District wise distribution of different* Trichoderma* and* Hypocrea* species in Manipur.

Out of the total isolates obtained from nine geographically distinct areas of Manipur, 65 isolates were preliminarily identified at the species level by morphological characteristics and later identified by internal transcribed spacer (ITS) sequences. Altogether 22 different* Trichoderma* species were identified as follows:* T. harzianum*,* H. rufa, H. intricate*,* T. aureoviride*,* T. atroviride*,* T. asperellum*,* T. amazonicum*,* T. caribbaeum*,* T. ovalisporum*,* T. inhamatum*,* T. tomentosum*,* T. longibrachiatum*,* T. koningiopsis*,* T. erinaceum*,* T. hamatum*,* Hypocrea virens*,* T. petersenii*,* T. gamsii*,* T. spirale*,* T. piluliferum*,* T. album,* and* H. nigricans*. The species wise distributions of* Trichoderma* isolates in 9 different districts of Manipur were represented in Figure 4. The identification, origin, and NCBI Genebank accession numbers and isolation details are given in Table 1.

### 3.4. Production of Different Cell Wall Degrading Enzymes

Each of the representative species of 65* Trichoderma* strains was determined for the production of different enzymatic activities, namely, chitinase, protease, and *β*-1,3-glucanase. These enzymatic activities for each one of the representative strains of 22 different* Trichoderma* spp. are given in Table 2. Chitinase enzyme that can degrade chitin, a major component of structural polysaccharide of the fungal pathogen cell wall, was evaluated using the chitin detection medium. The diameter of the purple color zone formation indicates the presence of chitinase activity and its zone diameter ranged from 41 to 79.66 mm in which T67 (*T. aureoviride*) showed the highest chitinase activity. The production of extracellular protease enzyme was determined for all the strains in skim milk agar plate. The diameter of halo zone formation ranged from 10.33 to 42.66 mm, in which T176 (*T. spirale*) was the highest producer of protease enzyme. *β*-1,3 glucanase which hydrolyzes the O-glycosidic linkages of *β*-glucan chains in the fungal cell wall is one of the important defense mechanisms exhibited by* Trichoderma* to fight against fungal pathogens. All the 22 representative strains of* Trichoderma* spp. exhibited *β*-1,3-glucanase activity with a clearance zone diameter ranging from 11.33 to 53.66 mm. T12 (*T. harzianum*) produced the highest *β*-1,3-glucanase enzyme activity.

### 3.5. Taxonomy

Various phenotypic differences were observed among the 22 investigated groups of* Trichoderma* spp. All the species were able to grow between 15°C and 30°C with different growth rates.* Trichoderma* species belongs to the division Ascomycota, subdivision Pezizomycotina, class Sordariomycetes, subclass Hypocreomycetidae, order Hypocreales, and family Hypocreaceae.

(I)* Trichoderma harzianum* Indira & kamala

*Synonyms*.* Trichoderma inhamatum* Veerkamp & W. Gams.

*Teleomorph*. Hypocrea lixii Patouillard.

**Figure 5:**
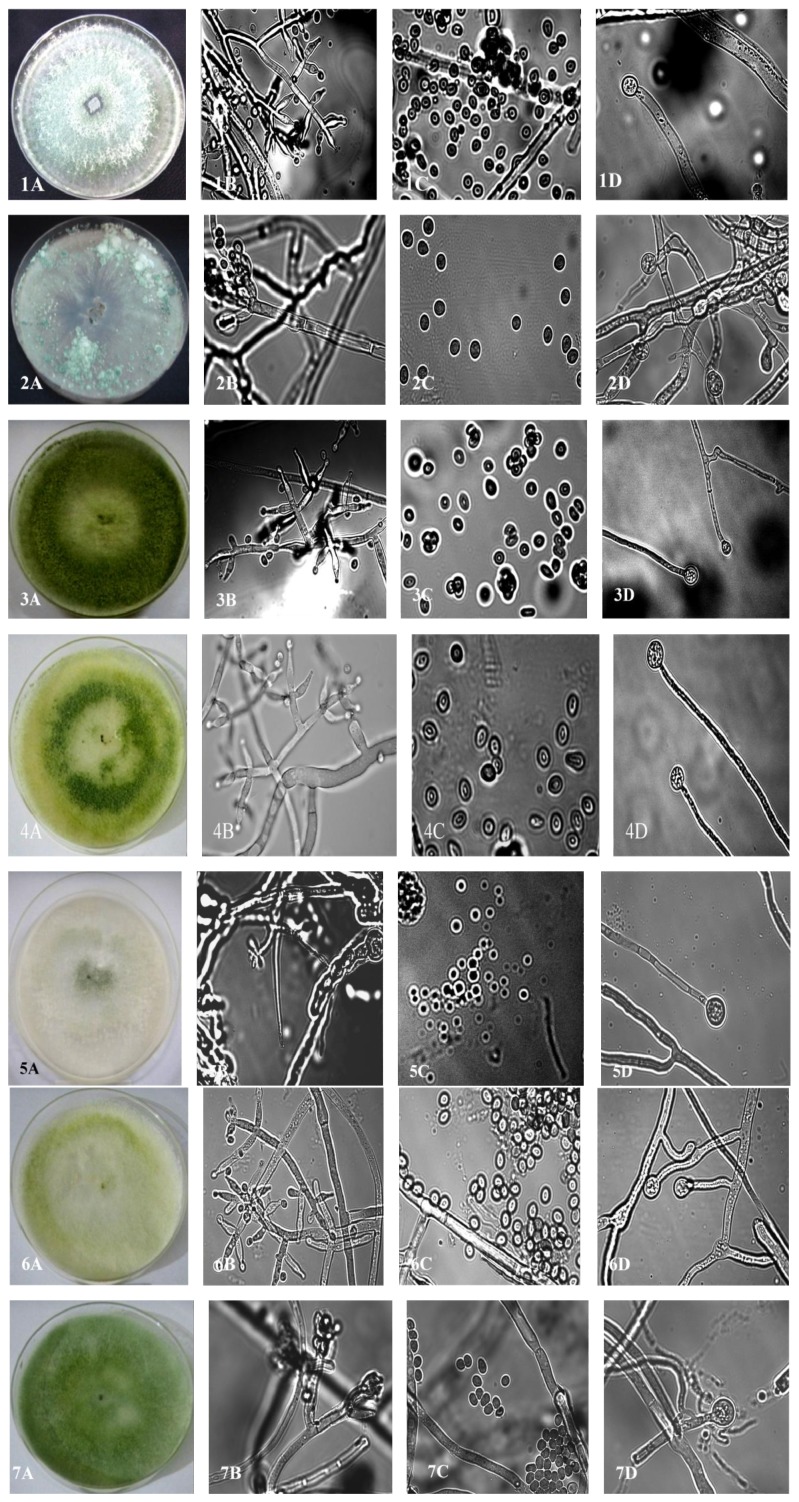
Morphology, conidiophore, spore, and chlamydospore of different* Trichoderma* strains represented in A, B, C, and D, respectively: 1:* T. harzianum*, 2:* H. rufa*, 3:* T. aureoviride*, 4:* T. atroviride*, 5:* H. intricata*, 6:* T. inhamatum*, and 7:* T. koningiopsis*.

*Diagnosis*. It is a slow growing species with green colony. Hyphae forms cottony white, with watery white in color, mycelia sparse and produced floccose aerial mycelium, conidiophore pyramidal with short vertical intervals and short base secondary branch, phialide ampulliform to flask-shaped (L/W 4.8–8.5 × 2.5–3.5 *μ*m), conidia (L/W 2.7–3.5 × 2.5–3 *μ*m) globose or subglobose, and no scar. Chlamydospores were produced in old cultures which were globose to subglobose, terminal or intercalary, stromata 1.0–1.5 mm diameter, surface smooth, solitary or aggregated, pulvinate, nearly circular in outline, ascospore (L/W 4.3-4.4 × 3.9 × 4.0 *μ*m) green in colour. Pigment often forms yellow color diffusing in medium and no distinct odor detected (Figure 5). 

*Optimum Growth Temperature*. 30°C.

*Optimum pH*. 7.00-8.00. 

*Extrolite*. Peptaibols, anthraquinones, and harzianopyridone. 

*Enzyme Production*. Chitinases, *β*-1,3-glucanase, protease.

(II)* Hypocrea rufa* Indira & kamala

*Synonyms*.* Sphaeria rufa *Persoon. 

*Anamorph*.* Trichoderma viride *Persoon

 =* Trichoderma lignorum* Tode Harz
 =* Trichoderma glaucum* Abbott

*Diagnosis*. They are fast growing species with whitish to tan to reddish brown and become darker and cushion-shaped after prolonged growth. Conidiophore conspicuous and extremely variable (curved to sinuous), phialide unpaired, L/W (1.2) 2.0–4.5 (−13.0) *μ*m, conidia L/W 1.0–1.4 *μ*m, pulvinate to hemispherical pustules (<1–3 mm). Chlamydospore not observed, stromata fleshy, ascospore hyaline, L/W 4.2–5.2 × 4.0–4.5 *μ*m, 2-celled fractured at the septum within asci, no distinct odor detected. No distinct pigments detected (Figure 5). 

*Optimum Growth Temperature*. 25°C–30°C. 

*Optimum pH*. 4.00. 

*Extrolites*. Peptaibols. 

*Enzyme Production*. Protease, chitinase, *β*-1,3 glucanase.

(III)* Trichoderma aureoviride* Indira & kamala

*Synonyms. Chromocrea aureoviridis* Plowr & Cooke. 

*Teleomorph*.* Hypocrea aureoviridis* Chaverri. 

*Diagnosis*. Fast growing with optimum growth temperature between 20°C to 25°C. Colonies uniformly flat and velvety, colony color cool white, yellow pigment produced. Hyphae form uniform lawn over the white colony, mycelia aerial comprising short hyphae in the form of a uniform lawn over the colony. Conidiophore arises from substrate hyphae or from aerial hyphae, 50–100 *μ*m long, smooth, typically branched along the length in a verticillate fashion. Conidia L/W 3.5–5 × 2.5–3 *μ*m, green, clavate to ellipsoidal or subglobose shape, often with a truncate or slightly protuberant base smooth, held in drops of pale green to colorless. Stromata solitary to gregarious, 1–5 mm diameter circular to elliptic in outline, centrally but broadly attached, at first yellow but becoming slightly rufous with age. Ascospore L/W 3.5–4.0 × 3.3–3.7 *μ*m, green, more or less monomorphic and subglobose, thick walled. Pigment intense yellow, no distinct odor detected (Figure 5). 

*Optimum Growth Temperature*. 20°C. 

*Optimum pH*. 4.00. 

*Extrolites*. Chrysophanol. 

*Enzymatic Production*. Protease, chitinase, *β*-1,3-glucanase.

(IV)* Trichoderma atroviride* Indira & kamala

*Synonyms*.* Trichoderma parceramosum* Bissett. 

*Teleomorph*.* Hypocrea atroviridis* Dodd. 

*Diagnosis*. Colony characteristics uniformly dispersed not pustulate or in conflict, sharply delimited and more or less dense central disk within which most conidia form, colony color green after sporulation, colony radius 42–60 mm in three days incubation. Hyphae white, sharply delimited with a more or less dense central disk within which most conidia form, mycelia formed uniform mat. Conidiophores branching typically unilateral although paired branches are common, branches typically arouse at 90° or less with respect to the branch above the point of branching, paired branching systems, phialide 6.0–9.7 *μ*m long, straight or sinuous, sometimes hooked, whorls of 2–4, often cylindrical and narrow neck, conidia L/W 1.0–1.3 *μ*m, subglobose to ovoidal, chlamydospores abundant within 7 days, globose to subglobose, terminal or intercalary, stromata L/W 0.9–2.4 *μ*m in diameter, solitary to gregarious, adjacent stromata often fused, ascospores L/W 4.3-4.4 × 3.9 × 4.0 *μ*m dimorphic, hyaline, thick walled, finely spinulose, distal part globose to subglobose, sweet (coconut) odor typically noticed (Figure 5). 

*Optimum Growth Temperature*. 25°C–30°C. 

*Optimum pH*. 4.00. 

*Extrolites*. Atroviridin. 

*Enzymatic Production*. *β*-1,3 glucanase, protease, chitinase.

(V)* Hypocrea intricata* Indira & kamala

*Anamorph*.* Trichoderma intricatum* Samuels and Schroers. 

*Diagnosis*. Colonies grow very fast filling the Petri plate within 1 week up to 90 mm diameter, dark green hyphae dense cottony with aerial mycelium, conidiophore formed around the margin of the colony in a more or less continuous, 2.0–4.0 *μ*m wide cottony pustules, with a discernible main axis, conidia abundant in the aerial mycelium formed concentric ring with dark green color, broadly ellipsoidal to ovoidal, smooth, phialides L/W 6.0–9.7 × 1.8–3.5 *μ*m, legeniform and somewhat swollen in the middle to cylindrical, straight, rarely slightly hooked or sinuous, stromata at first semieffused, 0.5–10 mm in diameter brownish orange to light brown with a white margin (0.5–10 mm diameter), chlamydospore not observed, ascospore L/W 4.3-4.4 × 3.9 × 4.0 *μ*m, hyaline, finely spinulate, dimorphic, distal part subglobose, proximal part wedge-shaped to oblong or slightly ellipsoidal. No diffusing pigments and no distinct odour were detected (Figure 5). 

*Optimum Growth Temperature*. 30°C. 

*Optimum pH*. 4.00. 

*Extrolites*. Peptaibols. 

*Enzyme Production*. *β*-1,3 glucanase, protease, chitinase.

(VI)* Trichoderma inhamatum* Indira & kamala

*Synonyms*.* Trichoderma harzianum* Rifai. 

*Teleomorph*.* Hypocrea lixii* Pat. 

*Diagnosis*. Colony cottony white at the beginning and later becoming light green after sporulation reaching up to 45–50 mm radius in four-day incubation. Mycelia completely or nearly filling the Petri dish, conidiophores 2.0–4.0 *μ*m wide narrow, flexuous, branches, lack of sterile appendages, phialide uncrowded, frequently paired, globose, chlamydospore not observed, conidia L/W 3.0–3.5 × 2.2–2.5 *μ*m, formed abundantly within 72 h at 25°–30°C on PDA. Yellow pigmentation produced and no odor detected (Figure 5). 

*Optimum Growth Temperature*. 25°C–30°C. 

*Optimum pH*. 4.00. 

*Extrolites*. Peptaibols. 

*Enzymatic Production*. *β*-1,3-glucanase, protease, chitinase.

(VII)* Trichoderma koningiopsis* Indira & kamala

*Teleomorph*.* Hypocrea koningiopsis* Samuels. 

*Diagnosis*. Colony color compact to cottony white at early stage, later forming deep green to dark green, seldom yellow coloration. Conidial production sometimes restricted to the margin of the colony, sometimes forming cottony pustules, colony radius up to 51–63 mm forming 2-3 concentric rings. Hyphae form dense lawn. Mycelia aerial with broad concentric rings, sometimes forming cottony pustules. Conidiophore branched with long internodes between branches, branches arising slightly less than 90° with respect to the main axis. Phialides L/W 5.5–9.0 × 1.3–3.3 *μ*m, straight, sometimes hooked or sinuous, narrowly lageniform or sometimes swollen in the middle, intercalary phialides present (5.5–9.0 *μ*m long). Conidia L/W 3.5–4.5 × 2.2–3.5 *μ*m deep green to dark green, seldom with yellow coloration, ellipsoidal, lacking a visible basal abscission scar, smooth (3.5–4.5 *μ*m) dry. Chlamydospores abundant to sparse, terminal to intercalary, globose to subglobose, 9.0–9.5 *μ*m diameter. Stromata scattered, circular in outline, 1.5–2.5 mm diameter, broadly attached, margins sometimes free, convex to plane, pulvinate. Ascospores L/W 3.7–4.7 × 2.2–3.5 *μ*m dimorphic, hyaline, thick walled, finely spinulose, distal part globose to subglobose (2.5–3.5 *μ*m), proximal part oblong to wedge-shaped (3.7–4.7 *μ*m) (Figure 5). 

*Optimum Growth Temperature*. 25°C–30°C. 

*Optimum pH*. 4.00. 

*Extrolites*. Peptaibols. 

*Enzyme Production*. Protease, chitinase, *β*-1,3-glucanase.

(VIII)* Hypocrea virens* Indira & kamala

*Anamorph*.* T. virens* Miller, Giddens & Foster

 =* Gliocladium virens* Miller
 =* Trichoderma flavofuscum* Miller
 =* Gliocladium flavofuscum* Miller

**Figure 6:**
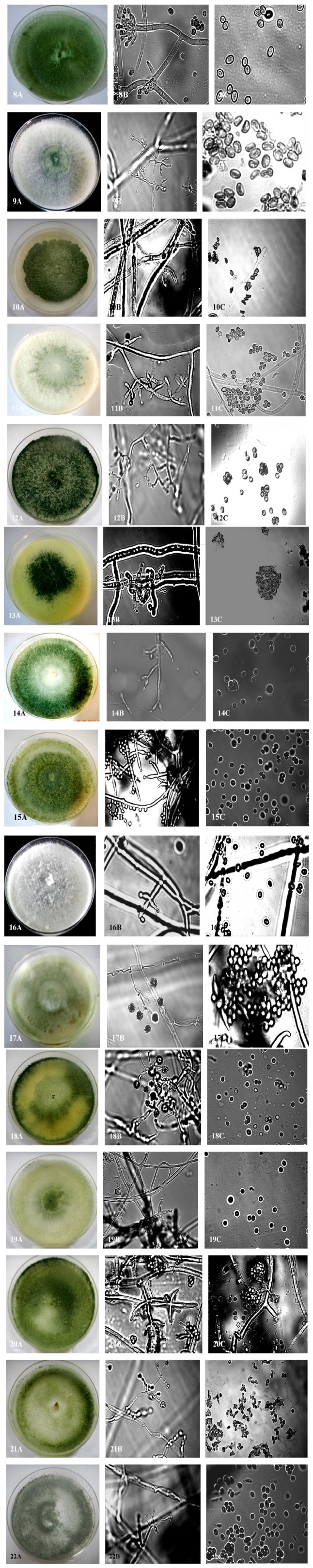
Morphology, conidiophore, spore, and chlamydospore of different* Trichoderma* strains represented in A, B, C, and D, respectively: 8:* H. virens*, 9:* T. album*, 10:* T. hamatum*, 11:* T. petersenii*, 12:* T. asperellum*, 13:* T. longibrachiatum*, 14:* T. caribbaeum*, 15:* T. amazonicum*, 16:* T. gamsii*, 17:* T. spirale*, 18:* T. tomentosum*, 19:* T. piluliferum*, 20:* T. ovalisporum*, 21:* H. nigricans, *and 22:* T. erinaceum.*

*Diagnosis*. Colony growing fast up to 90 mm diameter within three-day incubation, floccose with effuse conidiation typically covering the entire surface of the plate, conidiophores hyaline, smooth-walled, L/W 12.4–133.0 × 4.2–6 *μ*m, conidia produced concentrically or near the margin of the plate, metulae (subtending hyphae) cylindrical (L/W 8.5–13.8 × 2.7–4.8 *μ*m), stromata 0.8–1.0 mm, solitary and scattered, pulvinate, light yellow, nearly circular in outline, phialides lageniform to ampulliform, length 8.6–9.9 *μ*m, base 2.1–2.7 *μ*m wide, width at the widest 3.4–4.5 *μ*m. Conidia green, smooth, broadly ellipsoidal to obovoid, 4.2–4.9 × 3.6–4.2 *μ*m. Chlamydospores abundant, terminal or intercalary, subglobose, 6.3–12.4 × 6.1–10.1 *μ*m. A yellow pigmentation of the agar was sometimes present on PDA (Figure 6).

*Optimum Growth Temperature*. 25°C–30°C. 

*Optimum pH*. 4.00. 

*Extrolites*. Peptaibols. 

*Enzyme Production*. Protease, chitinase, *β*-1,3-glucanase.

(IX)* Trichoderma album* Indira & kamala 

*Synonyms*.* Trichoderma polysporum *Rifai. 

*Diagnosis*. Fast growing with uniform spreading mycelia and color changing to light green, after sporulation, hyphae somewhat cottony, green conidia forming in thick and broadly ellipsoidal, mycelia aerial, branched conidiophore, phialide somewhat swollen in the middle. No distinct pigments formed and no distinct odour was detected (Figure 6). 

*Optimum Growth Temperature*. 25°C–30°C. 

*Optimum pH*. 4.00. 

*Extrolites*. Peptaibols. 

*Enzymatic Production*. Protease, chitinase, *β*-1,3-glucanase.

(X)* Trichoderma hamatum* Indira & kamala

*Synonyms*.* Verticillium hamatum* Bonorden

 =* Pachybasium hamatum* Bonord. Saccardo
 =* Phymatotrichum hamatum* Bonorden
 =* Monosporium ellipticum* Daszewska.

*Diagnosis*. Colony grew moderately, reaching up to 5 cm in diameter after 3-day incubation, very white and often grew densely, producing some aerial mycelium which is fluccose in nature, produce disperse cushion shaped. Hyphae white with dense central disk, mycelia grew mostly close to the agar, conidiophore compact tufts with large color variation, pale yellow and greenish yellow to greyish green, phialides densely clustered on wide main axis, conidia L/W 4.2–5.0 × 2.7–3.0 *μ*m, green ellipsoidal, 2.7–3.0 *μ*m, smooth, chlamydospores terminal and intercalary, subglobose to globose with 10–13 *μ*m diameter, 48–53 mm colony radius at 25°C–30°C, not growing at or above 35°C. No diffusing pigments and no odor produced (Figure 6). 

*Optimum Growth Temperature*. 25°C–30°C. 

*Optimum pH*. 6.5. 

*Extrolites*. Dermadin. 

*Enzymatic Production*. *β*-1,3-glucanase, chitinase, protease.

(XI)* Trichoderma petersenii* Indira & Kamala

*Teleomorph*.* Hypocrea petersenii* Samuels. 

*Diagnosis*. Colony formed conspicuous concentric ring, colony diameter 33–45 mm, colony typically formed abundant conidia with concentric rings, conidia formed dark green color. Hyphae cottony white, mycelium aerial, conidiophore often visible in pustules, entirely fertile and plumose, symmetrical, comprising a recognizable main axis. Phialides L/W 8.8–9.2 × 4.0–4.2 *μ*m, typically straight, legeniform, cylindrical or slightly swollen in the middle, held in whorls of 3 to 4, intercalary phialides not seen, conidia L/W 4.2–5.0 × 2.7–3.0 *μ*m, ellipsoidal to broadly ellipsoidal, smooth. Chlamydospore abundant to sparse or lacking, terminal or intercalary, globose to subglobose (6.5–12 mm diameter). Stromata 0.8–1.0 mm, scattered to gregarious, at first thin, semieffused, tan with a lighter-coloured margin, velvety, gradually becoming thicker, pulvinate to discoidal and reddish brown. Ascospores L/W 3.0-4.0 × 2.7–3.7 *μ*m, hyaline, finely spinulose, dimorphic, distal part subglobose, proximal part wedge-shaped to oblong or slightly ellipsoidal. No pigment formed and no distinctive odor detected (Figure 6). 

*Optimum Growth Temperature*. 25°C–30°C. 

*Optimum pH*. 4.00. 

*Extrolites*. Peptaibols. 

*Enzyme Production*. Protease, *β*-1,3-glucanase, chitinase.

(XII)* Trichoderma asperellum* Indira & Kamala 

*Teleomorph*.* Hypocrea asperella* Starback. 

*Diagnosis*. Colony grew moderately forming up to 5 concentric rings of dense conidial production, hyphae formed lawn, mycelia sparse and grew close to the agar, aerial mycelium lacking, conidiophore regularly branched and typically paired, phialide straight, conidia L/W 1.0–1.7 *μ*m, green to dark green, cushion shaped tufts, subglobose or ovoidal, finely spinulose. Chlamydospore abundant within one week, terminal or infrequently intercalary, hyphae, subglobose to ovoidal, smooth, and pale green. No distinct pigments and no distinct odour were detected (Figure 6). 

*Optimum Growth Temperature*. 30°C. 

*Optimum pH*. 4.00. 

*Extrolites*. Peptaibols. 

*Enzyme Production*. Protease, chitinase, *β*-1,3-glucanase.

(XIII)* Trichoderma longibrachiatum* Indira & Kamala

*Teleomorph*.* H. orientalis* Samuels. 

*Diagnosis*. Colony continuous, confluent pulvinate aggregates, colony radius 65–70 mm within three days of incubation, conidial mass dark green, sometimes mottled with white flecks, conidia formed within 24 h at 30°–40°C tending to form concentric rings, hyphae sometimes mottled with white flecks and often with inconspicuous wefts of yellow hyphae on the surface of the conidial mass. Conidiophore consists of a strongly developed central axis, often paired; the main axis was 2.2–3.2 *μ*m wide, phialides L/W 4.8–8.5 × 2.5–3.5 *μ*m, solitary, rarely in verticils, intercalary. Conidia ellipsoidal to oblong, green in color. Chlamydospores generally abundant, terminal and then subglobose to globose or intercalary. Pigment yellow diffusing through the agar, no distinct odor detected (Figure 6). 

*Optimum Growth Temperature*. 30°C–35°C. 

*Optimum pH*. 6.5. 

*Extrolites.* Peptaibols. 

*Enzyme Production*. Protease, chitinase, *β*-1,3-glucanase.

(XIV)* Trichoderma caribbaeum* Indira & Kamala

*Teleomorph*.* Hypocrea caribbaea* Samuels & Schroers. 

*Diagnosis*. Colony radius 40–45 mm, green after sporulation with faint concentric rings or poor conidial production, conidia formed slowly on PDA, after 72–96 h at 20°C, hyphae uniform cottony, mycelia abundant aerial, conidiophore projecting from the pustules, entirely fertile or sparingly branched, phialides L/W 6.4–7.5 × 3.1–3.4 *μ*m, held in cruciate to verticillate whorls of 3 or 4 arising singly, straight, lageniform, somewhat swollen in the middle, conidia L/W 3.1-3.2 × 3 *μ*m, green without yellow coloration, ellipsoidal to nearly oblong, smooth. Chlamydospore produced sparingly, terminal on hyphae, subglobose, stromata 0.5–10 mm in diameter, scattered, light brown to brownish orange, irregular or nearly circular, more or less pulvinate, ascospore L/W 4.2–5.2 × 4.0–4.5 *μ*m, cylindrical, apex thickened, with a minute pore, hyaline, finely spinulose, dimorphic, distal part subglobose to slightly conical. No pigment diffusing through the agar and no distinct odor detected (Figure 6). 

*Optimum Growth Temperature*. 25°C–30°C. 

*Optimum pH*. 4.00. 

*Extrolites*. Peptaibols. 

*Enzyme Production*. Protease, chitinase, *β*-1,3-glucanase.

(XV)* Trichoderma amazonicum* Indira & Kamala

*Teleomorph. Hypocrea amazonica* Cooke. 

*Holotypes*. Cultura sicca BPI880413, culturia viva IB 50. 

*Etymology*. “Amazonicum” for its origin in the Amazon basin. 

*Diagnosis*. Colony forms cottony, colony radius reached 59–69 mm at 25°C, green conidia formed in thick and dense concentric rings, colony formed loose pustules, tending to aggregate towards the distal parts of the colony, few aerial hyphae, mycelia completely filling the Petri dish with dense conidia. Conidiophore pyramidal fashion, branches arise at almost 90° with respect to the main axis, phialide flask-shaped (6.4–7.7 × 3.3–3.5 *μ*m), conidia (3.2–3.4 × 3, L/W 1.2–1.20), globose, scar generally visible, chlamydospore-like structures formed in clusters, hyaline and thin-walled. No diffusing pigmentation, slightly fruity odor found (Figure 6). 

*Optimum Growth Temperature*. 25°C–30°C. 

*Optimum pH*. 4.00. 

*Extrolites*. Peptaibols. 

*Enzyme Production*. Protease, chitinase, *β*-1,3-glucanase.

(XVI)* Trichoderma gamsii* Indira & Kamala

*Diagnosis*. Colony characteristics at first yellow, then gradually changing to green, pustules cottony to dense, conidia forming abundantly conspicuous concentric rings, dense white, colony diameter (>45 mm after 72 hr). Hyphae forming mat-like structure. Conidiophore neither extensive nor uniformly branched, phialides L/W 8.5–9.0 × 4.0–4.2 *μ*m, solitary, common, lageniform. Conidia L/W 3.2–5 × 2.5–3 *μ*m, smooth, ellipsoidal, and warted in structure. Chlamydospores typically abundant, subglobose, terminal hyphae (8.0–11.5 *μ*m), sometimes with a pale yellow diffusing pigment, typically with a strong coconut-like odor (Figure 6). 

*Optimum Growth Temperature*. 25°C–30°C. 

*Optimum pH*. 4.00. 

*Extrolites*. Peptaibols. 

*Enzyme Production*. Protease, chitinase, *β*-1,3-glucanase.

(XVII)* Trichoderma spirale* Indira & Kamala

*Diagnosis*. Colony formed more or less distinct concentric rings. Pustules typically formed, pulvinate to subglobose, gray-green (0.5–1.5 mm), compact, colony color yellowish green. Hyphae formed uniform lawn, mycelia cool white fluorescent light with pustules formed around the periphery of the colony and a synanamorph forming abundantly in the aerial mycelium. Conidiophore formed a sterile hair from the base from which arise short, broad fertile branches. Phialides L/W 8.8–9.2 × 4.0–4.2 *μ*m, arise singly, directly from any of the branches, or they arise in whorls at the end of branches, phialides often doliform then clustered in grape-like fashion, when not densely clustered they are ampulliform. Conidia L/W 3.5–4.5 × 2.5–3.0 *μ*m, green in color, oblong to narrowly ellipsoidal (2.5–3.0 *μ*m), smooth. Chlamydospores typically abundant, intercalary, often formed in chains of several globose to subglobose (7.0–15.0 *μ*m) diameter. A yellow pigment tending to diffuse through the agar within 48 h (Figure 6). 

*Optimum Growth Temperature*. 30°C. 

*Optimum pH*. 4.00. 

*Extrolites*. Peptaibols. 

*Enzyme Production*. Protease, chitinase, *β*-1,3-glucanase.

(XVIII)* Trichoderma tomentosum* Indira & Kamala

*Synonyms*.* Trichoderma cerinum.*

*Diagnosis*. Fast growing, conidia first appeared within 72 h at 25–30°C in a dense central disk, after 144 h conidia formed into pronounced concentric rings. Colony formed pustules in a narrow band around the edge of the colony, colony cool white fluorescent light, colony radius reached up to 45–50 mm. Hyphae dense, mycelia cool white, conidia formed pustules in the narrow band around the edge of the colony, synanamorph abundant in the aerial mycelium, conidiophore comprised an unbranched or infrequently branched sterile hair from the base, phialide L/W 4.5–5.5 × 2.8–3.5 *μ*m, tending to be short and broad, almost ovoidal with a distinct neck and grape-like clusters at the tips of fertile branches, conidia L/W 3.0–3.5 × 2.2–5.2 *μ*m, grey-green, broadly ellipsoidal, smooth, chlamydospore scattered on CMD, globose to subglobose, terminal or intercalary. No diffusing pigment and distinctive odour detected (Figure 6). 

*Optimum Growth Temperature*. 25°C–30°C. 

*Optimum pH*. 4.00. 

*Metabolite Production*. Peptaibols. 

*Enzyme Production*. protease, chitinase, *β*-1,3-glucanase.

(XIX)* Trichoderma piluliferum* Indira & Kamala

*Teleomorph*.* Hypocrea pilulifera* Lu.

*Diagnosis*. Fast growing, initially formed white colony and subsequently green, colony diameter reaching up to 40–50 mm, colony rough in nature, olive green color in later stages. Hyphae and mycelia rough and spiny. Conidiophore more or less symmetrical near the tip, branches arising at 90°. Phialides L/W 8.8–9.2 × 4.0–4.2 *μ*m, botryose arrangement of plumbroad phialides on a broad conidiophore. Conidia L/W 4.3–4.5 × 3.6–4.0 *μ*m, hyaline, smooth and rough, rounded, globose, sometimes with papilla. Chlamydospores terminal and intercalary in chain (Figure 6). 

*Optimum Growth Temperature*. 25°C–30°C. 

*Optimum pH*. 4.00. 

*Extrolite*. Peptaibols. 

*Enzyme Production*. Protease, chitinase, *β*-1,3-glucanase.

(XX)* Trichoderma ovalisporum* Indira & Kamala

*Synonyms*.* Trichoderma koningii.*

*Diagnosis*. Fast growth, colony characteristics cottony having grey-green pustules, each pustule comprising intertwined hyphae, phialides and conidia, colony color intermittent light forming green conidia, conidia ovoidal to broadly ellipsoidal or subglobose, L/W 1.1–1.3 (−1.6), formed abundantly in faint concentric rings within 72 h at 25°C–30°C on PDA, hyphae 3.0–4.5 mm, mycelia nearly or completely filling the Petri dish, confluent green pustules forming in the centre of the colony, conidiophore conspicuous, arising at or near 90° with respect to the main axis, secondary branches producing phialides directly, main axis ranging from 1.7–2.0 to 4.0−6.2 *μ*m wide, phialides paired or arising in whorls, typically arising at 90° with respect to the cell below, flask-shaped, intercalary phialides formed, chlamydospore: scattered, subglobose, terminal in submerged hyphae, L/W (6.0–) 7.0–10.0 (−12) × (4.0–) 6.2–8.7–10.5 *μ*m. No pigment and no odor detected (Figure 6). 

*Optimum Growth Temperature.* 25°C–30°C. 

*Optimum pH*. 4.00. 

*Extrolites*. Peptaibols. 

*Enzyme Production*. Protease, chitinase, *β*-1,3-glucanase.

(XXI)* Hypocrea nigricans* Indira & Kamala

*Synonyms*.* Hypocrea lentiformis*, Rehm 1898,* Chromocrea nigricans* S. Imai 1935. 

*Diagnosis*. Colony radius 45–50 mm formed pustules in culture, conidiophore aggregated forming weakly developed pustules, phialides straight, 7.6 *μ*m length, 2.0 *μ*m width at the base, 2.8 *μ*m width at the widest shape, conidia 3.2 × 2.8 *μ*m (L/W), globose to subglobose, chlamydospore: 7.8 × 9.0 *μ*m. Pigment yellow, distinct odor not detected (Figure 6). 

*Optimum Growth Temperature*. 30°C. 

*Optimum pH*. 4.00. 

*Metabolites*. Peptaibols. 

*Enzymatic Production*. Protease, chitinase, *β*-1,3-glucanase.

(XXII)* Trichoderma erinaceum* Indira & Kamala

*Diagnosis*. Colony formed flat lawns in concentric rings with some tendency to form flat pustules reaching colony radius 60–65 mm within 3-day incubation and dark green in color. Hyphae formed flat lawns, mycelia concentric rings with some tendency to form flat pustules, conidiophore branches arising at angles of 90°C or less with respect to the main axis, the main axis of the conidiophore (2.2–3.0 *μ*m wide). Phialides arose from branches near the base or in whorls of 2 or 3, nearly cylindrical to swollen in the middle (6.0–8.0 *μ*m long), conidia 1.3–1.5 (L/W), ellipsoidal to broadly ellipsoidal (1.3–1.5), smooth, sometimes yellow associated with conidia in pustules. Chlamydospore terminal to intercalary, globose to subglobose (10.0–13.0 *μ*m). No diffusing pigment was detected. More or less strong odour detected (Figure 6). 

*Optimum Growth Temperature*. 25°C–30°C. 

*Optimum pH*. 4.00. 

*Extrolites*. Peptaibols. 

*Enzyme Production*. Protease, chitinase, *β*-1,3-glucanase.

## 4. Discussion

The present study on the occurrence and diversity of* Trichoderma* spp. from Manipur was carried out for the first time from this region. The samples were collected from different subtropical agroclimatic zones comprising both hills and plains and were allowed to grow in* Trichoderma* specific media. Despite the fact that* Trichoderma* spp. are major group of organisms found from the mycoflora in tropical forest and cultivated soil, their actual distribution, presence, and association with different plants and soils have not been fully investigated. The results from this study stress the importance of the use of molecular identifications tools to describe the occurrence of* Trichoderma* diversity from this region. Detection of polymorphism using PCR-RFLP analysis of the rDNA ITS region has been successfully used for identifying several species of fungi [38, 39]. In this study, from the total 193* Trichoderma* isolates obtained from 9 different districts, 65* Trichoderma* isolates were selected according to their morphological identification and they were grouped using ITS-RFLP using three restriction enzymes, namely, Taq1, Mob1, and Hindf1. The ITS region sequences data of the 65* Trichoderma* isolates gave 22 different species which were represented by both* Trichoderma* and* Hypocrea* spp. [40]. The bulk of evidence [41] strongly suggests that* Trichoderma* (anamorph) and* Hypocrea* (teleomorph) are a single holomorph genus. The result obtained from phylogenetic analysis of ITS sequences of 65* Trichoderma* isolates showed 4 distinct clades. Considering the phylogenetic analysis based on ITS sequences,* T. harzianum* represents the dominant group of* Trichoderma* spp. reported from this region [9, 42–44].

The 22 different representative* Trichoderma* spp. were* H. rufa, H. intricate, T. atroviride, T. asperellum, T. amazonicum, T. caribbaeum, T. ovalisporum, T. inhamatum, T. tomentosum, T. longibrachiatum, T. koningiopsis, T. erinaceum, T. hamatum, H. virens, T. petersenii, T. gamsii, T. spirale, T. piluliferum, T. album*, and* H. nigricans*. In this study, we also detected a remarkable diversity of genetically sibling species from the* Harzianum* clade in nearly all soil samples [45, 46]. The second abundant species identified in the present study was* Trichoderma aureoviride*. Major interrelated factors affecting microbial diversity in soil include physicochemical properties of soil. The relative effects of these factors differ in different soil types, horizons, and climatic zones [46]. The diversity and occurrence of* Trichoderma* species reported from four different agroclimatic zones of Manipur, namely, (i) subtropical plain zone, (ii) subtropical hill zone, (iii) temperate sub-Alpine zone, and (iv) midtropical hill zone, clearly indicate that the climatic topography and soil type are a major factor in the species distribution of* Trichoderma*. The subtropical plain zone comprises four districts, namely, Imphal East, Imphal West, Thoubal, and Bishnupur district. A total of 11 different species of* Trichoderma* occurred in this zone, namely,* T. atroviride, T. ovalisporum, T. album, T. tomentosum, T. harzianum, H. intricata, T. hamatum, H. rufa, T. aureoviride, T. inhamatum,* and* T. amazonicum,* and common soil types which occur in this region were alluvial, clay loamy, red gravelly sandy, and loamy soil. Subtropical hill zone covers two main districts, namely, Churachandpur and Chandel. In this district a total of 10 different types of Trichoderma species were found to occur, namely,* T. harzianum, T. longibrachiatum, T. piluliferem, T. petersenii, H. virens, H. rufa, H. nigricans, T. aereoviride, T. koningiopsis*, and* T. erinaceum,* with the occurrence of alluvial and loamy soil types. The temperate sub-Alpine zone comprises two districts, namely, Senapati and Ukhrul. In this type of zone only few types of* Trichoderma* variety were present, namely,* T. harzianum* and* T. aureoviride,* having soil type of lateritic black regur and red ferruginous, whereas, the midtropical hill zone covered only one district, namely, Tamenglong district, part of Ukhrul, and some portion of Imphal and Tamenglong district with the report of occurrence of 5 types of species, namely,* T. atroviride, T. album, T. koningiopsis, T. gamsii,* and* T. spirale*. The soil type mainly comprises alluvial soil. The occurrence of highest genetic diversity of* Trichoderma* species was reported from Bishnupur district with a total of eleven types of* Trichoderma* spp. followed by Chandel district with nine spp. and Tamenglong and Imphal East districts with five and four spp., respectively.

All the 22 representative strains of* Trichoderma* were found to produce three important enzymes, namely, chitinase, protease, and *β*-1,3-glucanase. The production of chitinase enzymes by these 22 representative strains which were represented by the purple color zone formation ranges from 41 to 79.66 mm in diameter. Harman et al. [3] described the types of chitinase detected from* T. harzianum*,* T. atroviride,* and* T. virens*. Howell [47] tested the role of chitinases in mycoparasitism and believed that chitinase is a key enzyme in this process. The protease activity ranged from 10.33 to 42.66 mm clearance zone diameter in skim milk agar medium. Benitez et al. [48] demonstrated that protease from* T. harzianum* plays an important role in biological control. Szekeres et al. [49] reported the role of protease in the mycoparasitism and have reinforced with the isolation of new protease overproducing strains of* T. harzianum*. *β*-1,3-glucanases have been found to be directly involved in the mycoparasitism interaction between* Trichoderma* species and its host [50]. Production of four *β*-1,3-glucanases and their role of hydrolyzing the O-glycosidic linkage of *β*-1,3-glucan chains in the fungal cell wall by* T. harzianum* have been described by Kitamoto et al. [51]. This work on diversity analysis of* Trichoderma* strains will provide a better identification of* Trichoderma* spp. with biocontrol mechanisms which can be used for the development of suitable bioformulation in sustainable agriculture.

## Figures and Tables

**Table 1:** Identification, origin, NCBI Genebank accession numbers, and isolation details of the 65 *Trichoderma* strains.

Sl. number	Isolation code	Species	Genebank accession number	Collection site	Source	Soil type	GPS location		
							Latitude	Longitude	Altitude (m)
1	IBSD T1	*T. harzianum *	JX518889	IW	Soil	Alluvial	25°00′N	94°15′E	790
2	IBSD T2	*H. intricata *	JX518890	IW	Soil	Alluvial	24°30′N	93°45′E	790
3	IBSD T8	*H. rufa *	JX518891	IE	Soil	Alluvial	23°55′N	92°59′E	790
4	IBSD T9	*H. rufa *	JX518892	IE	Soil	Alluvial	24°30′N	93°50′E	790
5	IBSD T10	*T. aureoviride *	JX518893	IE	Soil	Alluvial	24°30′N	93°50′E	790
6	IBSD T11	*T. harzianum *	JX518894	IE	Soil	Alluvial	23°55′N	92°59′E	790
7	IBSD T12	*T. harzianum *	JX518895	IE	Soil	Alluvial	92°50′E	23°55′N	790
8	IBSD T15	*T. atroviride *	JX518896	IE	Soil	Alluvial	23°30′N	93°54′E	787
9	IBSD T17	*T. harzianum *	JX518897	TH	Soil	Clay loamy	24°45′N	93°45′E	790
10	IBSD T20	*H. rufa *	JX518898	IE	Soil	Alluvial	23°56′N	92°59′E	795
11	IBSD T21	*T. album *	JX465711	TH	Soil	Clay loamy	93°45′E	23°45′N	790
12	IBSD T22	*T. atroviride *	JX465709	IE	Soil	Alluvial	24°30′N	93°50′E	790
13	IBSD T34	*T. inhamatum *	JX465706	B	Soil	Red gravelly sandy	23°45′N	93°45′E	790
14	IBSD T35	*T. aureoviride *	JX518899	B	Soil	loamy	24°30′N	93°45′E	790
15	IBSD T36	*T. aureoviride *	JX518900	B	Soil	loamy	24°30′N	93°45′E	790
16	IBSD T37	*T. inhamatum *	JX465705	B	Soil	Alluvial	24°15′N	94°15′E	790
17	IBSD T38	*T. inhamatum *	JX465703	B	Soil	Alluvial	24°30′N	93°45′E	790
18	IBSD T39	*T. asperellum *	JX518901	B	Soil	loamy	24°0′N	93°15′E	914.4
19	IBSD T40	*T. harzianum *	JX518902	B	Soil	Red gravelly sandy	24°44′N	93°78′E	828.18
20	IBSD T41	*T. amazonicum *	JX518903	B	Soil	Alluvial	92°59′E	23°55′N	790
21	IBSD T47	*T. harzianum *	JX518904	B	Soil	loamy	24°44′N	93°78′E	828.18
22	IBSD T54	*T. harzianum *	JX465708	B	Soil	loamy	24°44′N	93°78′E	828.18
23	IBSD T61	*T. atroviride *	JX465707	B	Soil	Alluvial	24°44′N	93°78′E	828.18
24	IBSD T62	*T. caribbaeum *	JX518905	B	Soil	Alluvial	24°37′N	93°29′E	1788
25	IBSD T66	*T. atroviride *	JX518906	B	Soil	Red gravelly sandy	24°44′N	93°78′E	828.18
26	IBSD T68	*T. aureoviride *	JX518907	B	Soil	Loamy	24°44′N	93°78′E	828.8
27	IBSD T69	*T. aureoviride *	JX518908	B	Soil	Alluvial	24°44′N	93°78′E	828.18
28	IBSD T70	*T. harzianum *	JX518909	B	Soil	Alluvial	24°44′N	93°78′E	828.18
29	IBSD T71	*T. ovalisporum *	JX518910	B	Soil	Alluvial	24°37′N	94°15′E	1061
30	IBSD T72	*T. aureoviride *	JX465701	B	Soil	Loamy	24°15′N	93°30′E	828.18
31	IBSD T74	*T. harzianum *	JX518911	B	Soil	Red gravelly sandy	24°44′N	93°78′E	828.18
32	IBSD T75	*T. harzianum *	JX518912	B	Soil	Alluvial	24°44′N	93°78′E	828.18
33	IBSD T77	*T. harzianum *	JX518913	B	Soil	Loamy	24°0′N	93°78′E	828.18
34	IBSD T78	*H. intricata *	JX518914	B	Soil	Red gravelly sandy	24°15′N	93°15′E	914.4
35	IBSD T80	*T. inhamatum *	JX518915	B	Soil	Alluvial	24°3′N	94°0′E	914.4
36	IBSD T81	*T. tomentosum *	JX518916	B	Soil	Alluvial	25°41′N	94°24′E	2113
37	IBSD T83	*T. atroviride *	JX518917	B	Soil	Loamy	24°0′N	94°0′E	1507
38	IBSD T85	*T. inhamatum *	JX518918	B	Soil	Red gravelly sandy	24°45′N	94°15′E	790
39	IBSD T86	*T. harzianum *	JX518919	B	Soil	Loamy	24°0′N	93°15′E	914.4
40	IBSD T88	*T. atroviride *	JX465700	B	Soil	Alluvial	24°44′N	93°78′E	828.18
41	IBSD T89	*T. harzianum *	JX518920	B	Soil	Alluvial	24°44′N	93°78′E	828.18
42	IBSD T100	*T. harzianum *	JX518924	S	Soil	Lateritic black regur	24°37′N	93°29′E	1061
43	IBSD T101	*T. harzianum *	JX518925	S	Soil	Red ferruginous	24°37′N	94°15′E	1561
44	IBSD T105	*T. aureoviride *	JX518926	U	Soil	Red ferruginous	24°44′N	93°15′E	914.4
45	IBSD T108	*H. nigricans *	JX465710	CH	Soil	Residual	24°0′N	93°15′E	914.4
46	IBSD T110	*T. longibrachiatum *	JX518921	CH	Soil	Transported	24°20′N	93°15′E	918
47	IBSD T112	*T. harzianum *	JX518927	U	Soil	Red ferruginous	24°30′N	94°47′E	3110
48	IBSD T114	*T. hamatum *	JX518928	IW	Soil	Alluvial	25°0′N	93°45′E	790
49	IBSD T116	*T. koningiopsis *	JX518922	C	Soil	Red gravelly sandy	24°40′N	93°50′E	787
50	IBSD T119	*T. aureoviride *	JX465702	C	Soil	Loamy	24°40′N	93°50′E	787
51	IBSD T121	*T. aureoviride *	JX518929	C	Soil	Loamy	24°40′N	93°50′E	790
52	IBSD T137	*T. harzianum *	JX518930	C	Soil	Alluvial	24°40′N	93°50′E	787
53	IBSD T142	*T. harzianum *	JX518931	C	Soil	Loamy	24°40′N	93°50′E	787
54	IBSD T149	*H. virens *	JX518932	C	Soil	Alluvial	24°40′N	93°50′E	790
55	IBSD T153	*T. erinaceum *	JX518923	C	Soil	Alluvial	24°37′N	93°29′E	1065
56	IBSD T155	*H. virens *	JX518933	C	Soil	Alluvial	24°40′N	93°50′E	787
57	IBSD T158	*H. rufa *	JX518934	C	Soil	Loamy	24°40′N	93°50′E	787
58	IBSD T161	*H.virens *	JX465699	C	Soil	Alluvial	24°40′N	93°50′E	790
59	IBSD T162	*T. petersenii *	JX518935	C	Soil	Loamy	24°45′N	94°15′E	795
60	IBSD T168	*T. album *	JX465704	T	Soil	Alluvial	24°59′N	93°30′E	1260
61	IBSD T174	*T. gamsii *	JX518936	T	Soil	Alluvial	24°59′N	93°30′E	1260
62	IBSD T176	*T. spirale *	JX518937	T	Soil	Alluvial	24°59′N	93°30′E	1265
63	IBSD T179	*T. piluliferum *	JX518938	C	Soil	Loamy	24°40′N	93°50′E	795
64	IBSD T184	*T. koningiopsis *	JX518939	T	Soil	Alluvial	24°59′N	93°30′E	1260
65	IBSD T186	*T. atroviride *	JX518940	T	Soil	Alluvial	24°59′N	93°30′E	1260

^*^Letters indicate the following locations: IW: soil samples from Imphal West district; IE: Imphal East district; TH: Thoubal district; B: Bishnupur district; S: Senapati district; U: Ukhrul district; CH: Churachandpur district; C: Chandel district, and T: Tamenglong district.

**Table 2:** Different enzymatic activity exhibited by 22 representative strains of *Trichoderma*.

Sl. number	*Trichoderma *	Chitinase (mm)	Protease (mm)	*β*-1,3-glucanase (mm)
1	*T. harzianum* (T12)	79.33 ± 0.66	23.33 ± 1.20	53.66 ± 0.66
2	*H. rufa* (T158)	70.33 ± 0.33	32.00 ± 1.52	31.33 ± 0.66
3	*H. intricatum * (T78)	69.66 ± 0.88	30.33 ± 0.33	39.33 ± 0.66
4	*T. aureoviride * (T67)	79.66 ± 2.02	30.33 ± 0.33	46.00 ± 0.57
5	*T. atroviride * (T22)	41.33 ± 0.88	12.33 ± 1.45	30.66 ± 0.66
6	*T. asperellum * (T39)	49.33 ± 0.66	27.33 ± 1.45	40.66 ± 0.33
7	*T. amazonicum * (T41)	47.66 ± 0.33	15.66 ± 0.66	39.00 ± 1.00
8	*T. caribbaeum * (T62)	70.33 ± 0.33	13.66 ± 0.66	41.66 ± 0.33
9	*T. ovalisporum * (T71)	70.33 ± 0.33	20.33 ± 0.33	30.33 ± 0.33
10	*T. inhamatum * (T37)	41.00 ± 0.57	14.66 ± 0.33	18.66 ± 1.85
11	*T. tomentosum * (T81)	72.66 ± 1.20	10.33 ± 0.33	32.33 ± 0.33
12	*T. longibrachiatum * (T110)	67.66 ± 1.45	20.66 ± 0.33	34.66 ± 1.33
13	*T. koningiopsis * (116)	77.33 ± 0.66	13.33 ± 1.20	38.66 ± 1.85
14	*T. erinaceum* (T153)	50.66 ± 0.66	11.00 ± 0.57	18.00 ± 1.00
15	*T. hamatum* (T114)	58.00 ± 2.51	29.00 ± 0.57	9.66 ± 0.33
16	*H. virens * (T149)	63.33 ± 1.66	29.66 ± 0.88	25.66 ± 0.33
17	*T. petersenii * (T162)	78.66 ± 0.66	37.33 ± 1.45	11.33 ± 0.66
18	*T. gamsii * (T174)	51.66 ± 1.66	31.00 ± 0.57	40.33 ± 0.33
19	*T. spirale * (T176)	78.33 ± 1.66	42.66 ± 1.45	34.66 ± 0.33
20	*T. piluliferum * (T179)	52.00 ± 1.52	31.00 ± 1.52	27.33 ± 0.66
21	*T. album * (T168)	60.33 ± 2.90	16.00 ± 0.57	41.00 ± 0.57
22	*H. nigricans * (T108)	63.00 ± 2.51	36.33 ± 0.33	51.66 ± 0.66
